# Reporting of Concussion Symptoms by a Nationwide Survey of United States Parents of Middle School Children

**DOI:** 10.3390/ijerph182212070

**Published:** 2021-11-17

**Authors:** Zachary Y. Kerr, Brittany M. Ingram, Christine E. Callahan, Aliza K. Nedimyer, Avinash Chandran, Melissa K. Kossman, Julia Hoang, Paula Gildner, Johna K. Register-Mihalik

**Affiliations:** 1 Department of Exercise and Sport Science, University of North Carolina, Chapel Hill, NC 27599-8700, USA; hoangjulia6@gmail.com (J.H.); pgildner@unc.edu (P.G.); johnakay@email.unc.edu (J.K.R.-M.); 2 Human Movement Science Curriculum, University of North Carolina, Chapel Hill, NC 27599-8700, USA; bmingram@live.unc.edu (B.M.I.); chriscal@live.unc.edu (C.E.C.); askamman@live.unc.edu (A.K.N.); 3 Datalys Center for Sports Injury Research and Prevention, Indianapolis, IN 46202, USA; avinashc@datalyscenter.org; 4 School of Health Professions, University of Southern Mississippi, Hattiesburg, MS 39406-0001, USA; melissa.k.kossman@usm.edu

**Keywords:** youth sports, recall, traumatic brain injury

## Abstract

This cross-sectional study assessed concussion symptom knowledge of parents of middle school (MS) children (aged 10–15 years) through a free-response item that solicited concussion symptoms and compared findings to a pre-validated scale-based measure. A self-administered online questionnaire was sent to a panel of randomly selected United States residents who were recruited by a third-party company, aged ≥ 18 years, and identified as parents of MS children. Via a free-response item, parents listed what they believed were concussion symptoms. Multiple sections later, parents identified potential concussion symptoms via a scale measure, which featured 25 items (22 actual symptoms, three distractor symptoms) with three response options: yes, no, maybe. Free-response item responses were coded into specific symptoms. The 1062 eligible parents that provided complete data commonly identified the symptoms of dizziness (90.2%), blurred vision (87.4%), and balance problems (86.4%) on the scale-based measure. However, these and other symptoms were less commonly identified via the free-response item (dizziness: 44.4%; blurred vision: 16.5%; balance problems: 3.5%). Concussion symptoms commonly reported via the scale-based measure were reported less frequently within the free-response item. Future research must explore strategies to help clinicians working with parents and their children to measure and assess concussion symptom reporting and knowledge.

## 1. Introduction

Concussions are reported in large numbers across youth populations within emergency department [1,2], healthcare [3], and sport settings [4,5,6]. Concerns regarding concussion may be further intensified given findings suggesting potential adverse outcomes associated with recurrent concussion and head impact exposure in current and former athletes [7,8,9,10]. Further, previous research estimates that 35–62% of concussions are not reported and thus, may not receive timely care [11]. As such, concussion is a public health issue that requires the development and implementation of interventions to reduce concussion incidence and severity and increase disclosure. 

Middle school is an intermediary level of education between elementary and secondary school that typically includes three to four grade levels (ranging from the 5th–9th grades) with students aged 10–15 years [4,12,13,14,15]. (Although this level may also be referred to as intermediate school or junior high, we will refer to it as middle school from here on). With a wide range of middle school athletes participating in various sports, preventing and treating potential injuries is important to maintain healthy, active youth athletes. Although middle school aged children may participate in a wide variety of sporting activities, middle school sports may be more representative of the broader population of children in the corresponding age group than youth sport leagues due to the lower costs and higher access for participation associated with middle school sports. Additionally, middle schools also include students who do not participate in sports, but may still be at risk for concussion due to other mechanisms such as falls and car crashes [3]. Given the young age of middle school students, parents are important and influential in the recognition of and response to concussions that are sustained by this population [16]. As such, understanding parents’ knowledge regarding concussion may help identify areas of need within concussion-related interventions [17].

Concussion knowledge assessment usually involves scale measures that assess an individual’s ability to identify specific signs and symptoms, (hereinafter simplified as “symptoms”) via checklists or true/false statements [12,18,19,20]. However, such measurements may be limited in that they may overestimate/artificially inflate knowledge due to two main factors: (1) providing symptoms that a respondent may not have previously considered; and (2) offering limited response options (i.e., two options for a true/false) that default a respondent to having a high probability of correctly responding to a prompt by random means [21]. Previous research has examined knowledge assessed via open-ended, free-response items [22,23,24,25], with a recent study of United States Air Force Academy cadets finding that colloquial terminology (e.g., “feeling loopy”) was sometimes used to describe concussion symptoms [25]. As work in the field progresses to develop and evaluate interventions that aim to increase concussion-related knowledge, it is important to consider how such knowledge is measured to ensure that valid measures can be acquired in order to better establish baseline knowledge and changes before and after intervention implementation. For parent populations, the inability to acquire valid measures may result in inaccurate conclusions regarding not only the effectiveness of such interventions, but also in how their knowledge levels are related to their decision-making regarding concussion care-seeking and management.

Given the methodological considerations noted, we examined data from a nationwide survey of parents of middle school-aged children (aged 10–15 years) that included two measurements of self-reported concussion symptom knowledge: first, a free-response item that solicited concussion symptoms; and second, a multi-item scale measure (i.e., symptom checklist) to assess concussion symptom knowledge. These data were used to explore two primary research questions (RQ) and one exploratory RQ:

RQ #1: What are the concussion symptoms reported by parents of middle school children (via a free-response item)?

RQ #2: How does knowledge of concussion symptoms compare when assessed via a free-response item versus a symptom checklist?

Exploratory RQ #3: How were parent- and child-related characteristics associated with the reporting of commonly noted concussion symptoms reported (via a free-response item)?

## 2. Materials and Methods

This study consisted of a cross-sectional survey completed by parents of middle school-aged children. The study was approved by the Institutional Review Board at the University of North Carolina at Chapel Hill. All participants provided informed consent prior to participation.

### 2.1. Participants

The population of interest was parents/legal guardians (here on referred to as “parents”) of middle school-aged children. The study sample was obtained from Survey Sampling International (SSI), which recruits a pool of US residents who have agreed to participate in online survey research. Upon recruitment, the participant pool provided demographic information that SSI used to identify those eligible for specific studies. SSI ensured data quality throughout the study through certification processes such as digital fingerprinting, IP verification, and built-in quality control questions.

For this study, only those who were US residents, aged ≥ 18 years, and reported being a parent of a child aged 10–15 years (the approximate age of middle school students) were included. No additional exclusion criteria were used. These eligibility criteria were shared with SSI, who randomly generated a sample from the eligible participant pool and invited them to participate in this survey study; participants were provided additional study information upon recruitment. At this point, SSI linked the eligible participant pool to the online questionnaire, which was hosted on Qualtrics (Qualtrics, Seattle, WA, USA). SSI reimbursed participants that completed the study with reward points that could be redeemed for cash, gift cards, or other incentives. The size of the SSI participant pool is unknown to the authors.

### 2.2. Data Collection

The Internet-based, self-administered questionnaire was based on a modified version of a previously validated instrument [26,27] including measures with high internal consistency [12]. Both the previous iteration and the current version were developed with input from parents of youth sport athletes, injury epidemiologists, athletic trainers, and other sports medicine professionals. The questionnaire was piloted in a sample of parents of middle school children and was subsequently revised to ensure clarity. The questionnaire captured information on characteristics of both the responding parents and their middle school children. The parent characteristics captured included: sex; age; race/ethnicity; highest level of education completed; and personal concussion history. The child characteristics (particular to their middle school child(ren)) captured included concussion history and participation in organized sports within the past year.

Our outcomes of interest were related to parent knowledge of concussion symptoms and originated from two items. First, parents were asked to respond to the following question in an open text, free-response-box: “In the space below, list what you think are the signs and symptoms of a concussion.” Later in the questionnaire, participants were also asked about concussion symptom knowledge via a scale measure, which featured 25 symptoms (22 actual symptoms and three distractor symptoms). Participants indicated whether they perceived a particular symptom to be concussion-related using the following response options: yes, no, maybe. Although it is possible that a parent may have been able to return to the free-response question to include symptoms potentially ascertained from the scale measure, this would have required going back multiple pages within the online platform.

### 2.3. Statistical Analysis

The first aspect of statistical analysis was to assess the free-response item related to potential concussion symptoms. A coder (JH) was trained to review parent responses and create categories based off responses. The coder and study lead (ZYK) met regularly to discuss coding and categorization. These regular meetings led to the creation of a codebook that evolved as coding ensued. The coder was not shown the symptom knowledge scale items to reduce potential bias favoring coding for those categories. If a parent noted multiple categories, the coder coded categories for each of those specific symptoms (e.g., “nausea, vomiting, headache, in some case unconsciousness” coded as “nausea/vomiting,” “headache,” and “loss of consciousness”).

At the beginning of the coding process, a check was conducted with 100 randomly-selected cases to assess agreement with the study lead and identify any additional training that might have been needed. Agreement was found to be high (Κ = 0.90) and coding continued. Upon completion of coding, the research team met to discuss broader groupings within which to fit potential symptoms. The broader groupings were based on the Institutes of Medicine, “Sports-Related Concussions in Youth,” document [28], and included: somatic, cognitive, emotional, and sleep. Additionally, based on previous research [29], vestibular-ocular symptoms were included as its own category. Resultant symptom groupings and codes were imported into the dataset and then analyzed using SAS (Version 9.4; SAS Institute Inc., Cary, NC, USA).

Parent and child characteristics were summarized using frequencies and percentages. For RQ #1 and RQ #2, which examined the concussion symptoms reported by parents via the free-response item and how they compared to the scale measure respectively, frequencies were calculated for each of the noted symptoms. For the scale measure, we assessed only the proportion of “yes” responses within the 22 real symptoms (but not the three distractor symptoms). To compare the identification of concussion symptoms via the free-response item versus the scale measure, we computed McNemar odds ratios (OR). McNemar’s test assesses the difference between two proportions, both of which derive from the same sample. The McNemar ORs indicated the discordance between each pair of measures from a parent. They are calculated based on the number of participants identifying a symptom on free-response item only (but not on the scale measure) divided by the number of participants identifying a symptom (i.e., responding “yes”) on the scale measure only (but not on the free-response item). Overall, ORs with 95% confidence intervals (CI) excluding 1.00 were deemed statistically significant.

For exploratory RQ #3, which examined how parent- and child-related characteristics were associated with the reporting of commonly-noted concussion symptoms reported via the free-response item, we first identified the ten most common symptoms reported. Next, with the ten most frequently noted symptoms, we used chi-square tests to compare the proportions of participants noting each symptom by: sex (male versus female); age (<40 versus ≥40 years, as this provided approximately half of the sample within each group); race (identified versus did not identify as White/non-Hispanic); highest level of education completed (less than bachelor’s degree versus bachelor’s degree or more); concussion history present for parent or child (yes versus no); and child played organized sports within the past year (yes versus no). An a priori alpha of 0.05 was used for these analyses.

## 3. Results

### 3.1. Sample Characteristics

During September and October 2018, 1362 participants agreed to complete the online questionnaire. Of these, 1292 (94.5%) met eligibility criteria. When asked to provide the symptoms of concussion via the free-response item, 207 did not provide a response and 23 could not be coded as answers were not serious or too vague (e.g., “pain”). These 230 participants were excluded from analyses, leaving 1062 included for analyses.

Most of the 1062 participants were female (65.7%), White/non-Hispanic (58.9%), and with less than a bachelor’s degree (55.4%; Table 1). The average age [±standard deviation (SD)] of the sample was 41 ± 10. Three-fourths (74.2%) noted their middle school-aged children participated in organized sports within the past year and 34.7% of participants noted either they or their middle school-aged children had a concussion history.

### 3.2. RQ #1: Concussion Symptoms Reported via Free-Response Item

The 1062 participants’ responses to the free-response item were coded. Fifty-three participants (5.0%) responded that they did not know any concussion symptoms. The remaining 1009 participants yielded 2983 specific responses representative of 70 symptoms (Table 2); an average of three symptoms were reported per parent (SD = 2; median (interquartile range): 3 (2–4); range: 1–17). Of the 2983 responses, 31.2% were categorized as *somatic* (31.2%), followed by *vestibular-ocular* (29.6%), and *cognitive* (27.2%). *Sleep* and *emotional* symptoms comprised 10.1% and 1.4%, respectively. The most commonly reported symptoms included: *headache* (49.5%); *dizziness* (44.4%); *nausea or vomiting* (28.0%); *confusion* (21.6%); *blurred vision* (16.5%); *fatigue or low energy* (14.2%); *difficulty remembering* (14.0%); *loss of consciousness* (14.0%); *eyes/pupils do not look right* (9.8%); and *feel sleepy* (9.2%; Table 2). Thus, overall, the 10 most reported symptoms included three *vestibular/ocular*, two *somatic*, three *cognitive,* two *sleep*, and zero *emotional symptoms.*

### 3.3. RQ #2: Differences in Concussion Symptoms Reported via Free-Response Item versus Scale Measure

Differences were evident when comparing concussion symptoms reported via the concussion symptom knowledge scale versus the free-response item (Table 3). The number of participants reporting each symptom was smaller via the free-response item versus the scale measure; also, the number of participants that identified a symptom on the free-response item only was smaller than that on the scale measure only. For example, dizziness (90.2%) was the most reported symptom on the scale measure, but was reported by only 44.4% of participants via the free-response item; 450 noted dizziness as a symptom in both measures; however, 22 and 508 participants noted dizziness as a symptom on the free-response item only and scale measure only, respectively. As highlighted by the McNemar OR, all symptoms had lower odds of being reported on the free-response item than on the concussion symptom knowledge scale.

### 3.4. Exploratory RQ #3: Differences in Concussion Symptoms Reported via Free-Response Item by Sample Characteristics

Overall, differences in the reporting of the ten most commonly reported symptoms varied by certain sample characteristics (Table 4). A higher number of differences were found when comparing proportions by sex, with 5 of the 10 symptoms having significant differences. Within these differences, we found higher proportions in women versus men in all but one symptom. For comparisons by age, differences were related to reporting *headache, dizziness, and eyes/pupils do not look right* as symptoms, with higher aged participants more likely to report. For comparisons by race/ethnicity, 5 of the 10 symptoms had significant differences, with all being more prevalently reported by those identifying as white/non-Hispanic. Less significant differences were reported when comparing by other sample characteristics, with no differences found for whether middle school-aged children had or had not participated in organized sports within the past year.

## 4. Discussion

This study used a large nationwide sample of parents of middle school children to examine what concussion symptoms were noted via a free-response item, and to compare free-response findings to that from a pre-existing scale measure. These findings suggest symptoms were more commonly identified as concussion symptoms within the scale measure as compared with the recall for free response. Findings highlight current scale-based measures with listed symptom recognition may result in an overestimate/artificial inflation of knowledge [21]. Continued examination of the development and implementation of concussion knowledge measures are needed to ensure valid measurements are acquired and the risk of misclassification (e.g., noting someone has high levels of knowledge when they do not, or vice versa) is minimized.

### 4.1. Concussion Symptoms Identified by Free-Response Item

A combination of vestibular/ocular, somatic, cognitive, and sleep symptoms were commonly noted via the free-response item. Findings are similar to previous research using samples of parents of youth athletes [22], college students [23], United States Air Force Academy cadets [25], and the general adult population [24], although specific proportions varied. As in those studies, our study found headache to be the most reported symptom in free response. Additionally, emotional-based symptoms were less likely to be noted, which parallels previous research inclusive of those using free-response and scale-based measures [17,23,30,31,32,33,34]. Gaps in concussion symptom knowledge are evident among parents and concussion educational interventions could highlight lesser-known symptoms. However, efforts to increase concussion knowledge may not inherently lead to being able to detect when a concussion has occurred [35]. It may also be difficult for parents to identify concussions sustained by their children as children may find it difficult to describe their symptoms [31]. Consequently, interventions aiming to help middle school parents increase concussion knowledge should also help them consider strategies to better respond to suspected concussions and access medical care to aid recovery [12]. This could also include helping children describe how they are feeling after an impact, particularly if they are unable to pinpoint specific symptoms, and helping parents better communicate concussion education and symptoms to their children [16].

### 4.2. Differences of Concussion Symptom Knowledge by Sample Characteristics

We also examined the free-response item in an exploratory research question focused on examining sample characteristic differences in the responses. As with previous studies that used both scale measures and free-response items [12,17,24,33,36,37], we observed differences within the sample, but mostly around age, sex, and race/ethnicity. In fact, both this study and previous research using the same sample but focused on the concussion symptom knowledge scale measure found differences by sex, age, and race/ethnicity [12]. Findings may further strengthen evidence suggesting differences in concussion symptom knowledge exist by these characteristics, regardless of how concussion symptom knowledge is ascertained (e.g., as a free-response item or scale measure). Moreover, findings substantiate calls for interventions that target specific sub-populations or address equity concerns by ensuring materials are accessible and representative of the cultural diversity within sport settings [12,18].

### 4.3. Comparisons between Concussion Symptom Free-Response Item and Scale Measure

Current study findings suggest symptoms were less identified through the free-response item compared to the scale measure. The proportion of participants identifying symptoms were considerably higher with the scale measure for all 22 symptoms considered. The resultant McNemar ORs were statistically significant, with mostly large or very large associations (i.e., OR < 0.11 and <0.03, respectively) [38]. These findings parallel a previous study assessing concussion symptom knowledge with both a free-response item and a checklist of 38 symptoms (including four distractor symptoms) [23]. Although an empirical evaluation of differences between both measures was not performed, lower proportions of participants were found to identify more commonly reported symptoms via the free-response item versus the scale measure. Such findings reiterate the knowledge gaps that exist among middle school parents and the need for interventions to help concussion recognition (more inclusive of actual recall) and response.

The findings between the two measures also highlight that the unprompted recall of concussion symptoms likely differs from recognition garnered via the matching of symptoms. With the former, middle school parents may struggle to identify all known symptoms, particularly when done through a multiple-question survey or interview. With the latter, symptoms provided in a list may have a high putative guess rate (e.g., 50% on a checklist or true/false and yes/no measures), and potential for acquiescence bias. As a result, scale measures may produce biased assessments that artificially inflate knowledge. This corresponds with previous research that has used scale measures and yielded highly skewed data in which the sample had high scores [12,33,39,40]. Thus, regardless of the method of concussion symptom recall used, caution must be taken in interpreting findings.

### 4.4. Methodological Implications

Scale measures for concussion symptom knowledge often include distractor symptoms not associated with concussion [12,17,40]. Despite this, participants may still register high knowledge scores simply for checking or selecting “Yes”/“True” for all possible symptoms (including the distractor symptoms). One possible solution is to include an equal number of real and distractor symptoms. Additionally, a progression to Likert-scale measures that better gauge participants’ level of certainty or confidence may also be warranted. However, it is important to note that we initially included such response items for our scale measure, but found in pilot-testing that parents were confused by the breadth of options and preferred our final measure using “Yes”, “No”, and “Maybe”.

Although utilized less frequently in research than a scale measure, a free-response item that allows participants to answer without being presented response options may offer a nuanced comprehension to how individuals recognize concussion-related symptoms [23]. Additionally, whereas a scale measure can identify overall knowledge, our approach with the free-response items (i.e., individual assessments of each symptom) may provide more nuanced details of where knowledge gaps exist in terms of specific symptoms or symptom domains (e.g., emotional, sleep). However, a free-response item still has limitations. A free-response item may be laborious in terms of coding and categorization. Additionally, in this study, 23 participants were excluded due to responses being too vague and unable to be interpreted (e.g., “pain”). Last, the identification of concussion symptoms via a scale measure or free-response item may differ from that in a real-world setting in which symptoms are experienced personally, are disclosed by their children, or witnessed (e.g., watching a hard hit during a child’s game).

Research has yet to fully explore the issues noted above or substantiate the solutions offered. It is important to evaluate these measures’ ability to ascertain knowledge with minimal bias. Further, the decision on which measure is most preferred for a particular study should be based upon numerous methodological and analytical aspects. These include considering whether the measure in question directly addressed the research question of interest; accounts for the needs and potential limitations of the use of a specific measure (e.g., measure may be hard to implement with a particular sample/population but more feasible with another); and is feasible with the available statistical power for data analysis.

### 4.5. Clinical Implications

Assessing parental knowledge of concussion may be important for clinicians, particularly those working with young athletes. Clinicians could consider adapting the simultaneous use of qualitative and quantitative symptom tracking methods (e.g., starting with questions focused on common symptoms, proceeding to use a checklist, and using responses from each to highlight symptoms identified and missed). As discussed in previous research [25], although the use of colloquial terms is often not recommended, education provided by clinicians may benefit from using such terminology used by patients and their caregivers to help them better identify concussion symptoms and thus, respond accordingly (e.g., seeking care). However, it is necessary to note that utilizing qualitative concussion symptoms assessments may burden clinicians and may not translate to impactful clinical application.

Furthermore, many middle school settings lack access to on-site athletic trainers and a school nurse may also be integral in providing education and care for children and their parents [41,42]. This is particularly important as previous research has found that, compared to high schools, middle schools were less likely to have policies related to concussion education for athletes, parents, and coaches [43]. Although the majority of concussion legislation accounts for middle school students [44], continued efforts are needed to ensure equitable access to education and care.

Last, parents are integral in responding to concussions sustained by youth [16]. Having knowledge of concussion symptoms will aid parents in recognizing suspected concussions and ensuring appropriate care and management. Previous research has suggested the role of parental influence on youth athletes’ concussion-related knowledge, although parental attitude, compared to parental knowledge, was found to be more associated [27]. However, such findings are limited and further examinations into parental influence and its application into concussion education efforts are warranted.

### 4.6. Limitations

Although our sample originated from a large, national participation pool, sampling bias is still possible. As such, our findings may not be generalizable to all middle school parents. When coding the free response qualitative data, some answers did not exactly match commonly reported symptoms making it difficult to be grouped and assigned to a symptom category. On the free-response item, middle school parents may have also not included all the symptoms they believed were associated with concussion and only those that were most common and/or recognizable. Using a free-response item followed by a related scale measure allows for parents to return to the free-response item and add in symptoms found on the scale measure that were not noted initially. However, doing so would have required significant effort. Lastly, our survey did not collect data on parents’ specific occupation, making it difficult to determine their exposure to concussion beyond reporting their personal and child’s concussion histories. Additional variables that may highlight differences, such as previously receiving concussion education or working in the medical or sport-related fields (e.g., physician, athletic trainer, coach), were not considered.

## 5. Conclusions

In line with previous studies in other populations, our study findings highlighted that concussion symptoms commonly reported via scale measures were reported at a lower prevalence with the free-response item among middle school parents. Findings suggested aids (such as the pre-existing list of symptoms included in scale-based measures) result in an overestimate/artificial inflation of knowledge. However, future research is necessary to explore strategies to best measure unbiased concussion symptom reporting and middle school parent concussion knowledge as this most parallels recall/recognition in real-world applications.

## Figures and Tables

**Table 1:** Characteristics of parents of middle school (MS) students (n = 1062).

Sample Characteristics	n	%
Sex		
Male	364	34.3
Female	698	65.7
Age (in years)		
Under 30	96	9.0
30–39	416	39.2
40–49	346	32.6
50 and over	204	19.2
Race/ethnicity		
White/non-Hispanic	626	58.9
Other racial/ethnic group	436	41.1
Hispanic/Latino	165	15.5
Black/African-American	140	13.2
Asian/Pacific-Islander	78	7.3
Mixed race/other	53	5.0
Highest level of education completed		
Less than bachelor’s degree	588	55.4
Less than high school	12	1.1
High school or GED	210	19.8
Some college, no degree	218	20.5
Associate’s degree	148	13.9
Bachelor’s degree or more	474	44.6
Bachelor’s degree	313	29.5
Master’s degree	108	10.2
Doctorate	24	2.3
Professional degree	29	2.7
Personal and child concussion history		
Neither	694	65.3
Personal history only	229	21.6
Child history only	68	6.4
Both personal and child history	71	6.7
MS child played organized sports within past year		
No	274	25.8
Yes	788	74.2

**Table 2:** Concussion symptoms reported by parents of middle school students (n = 1062) via free-response item ^1^.

Symptom Category and Symptoms	Participants, n (%)	Symptom Category and Symptoms	Participants, n (%)
**Somatic**		**Vestibular-ocular**	
Headache ^2^	526 (49.5)	Dizziness ^2^	472 (44.4)
Nausea or vomiting ^2^	297 (28.0)	Blurred vision ^2^	175 (16.5)
Sensitivity to light ^2^	34 (3.2)	Eyes/pupils do not look right	104 (9.8)
Bump on head	15 (1.4)	Balance problems ^2^	37 (3.5)
Head/brain swelling	10 (0.9)	Hearing changes	31 (2.9)
Neck pain ^2^	7 (0.7)	Cannot walk, get up, stand properly	16 (1.5)
Hard to breathe	6 (0.6)	See weird things	15 (1.4)
Chills and sweating	5 (0.5)	Not coordinated	14 (1.3)
Bruise on head	5 (0.5)	Shaky or wobbly	9 (0.8)
“Pressure in the head” ^2^	4 (0.4)	Motor issues	7 (0.7)
Sensitivity to noise ^2^	4 (0.4)	Blinking a lot	1 (0.1)
Numbness	4 (0.4)	Ear pain	1 (0.1)
Change in appetite	4 (0.4)	**Sleep**	
Seizure	4 (0.4)	Fatigue or low energy ^2^	151 (14.2)
Death	3 (0.3)	Feel sleepy	98 (9.2)
Change in heart rate	2 (0.2)	Drowsiness ^2^	31 (2.9)
**Cognitive**		Sleep disruptions	7 (0.7)
Confusion ^2^	229 (21.6)	Trouble waking up	4 (0.4)
Difficulty remembering ^2^	149 (14.0)	Sleep too much	4 (0.4)
Loss of consciousness	149 (14.0)	Restless	4 (0.4)
Difficulty speaking	96 (9.0)	Sleep too little	2 (0.2)
Disoriented	66 (6.2)	**Emotional**	
“Glazed over” look	33 (3.1)	Irritability ^2^	7 (0.7)
Not focused/scattered thoughts	26 (2.4)	Aggression/angered	7 (0.7)
Incoherent	14 (1.3)	Mood swings	7 (0.7)
Feeling like “in a fog” ^2^	12 (1.1)	Nervous or anxious ^2^	5 (0.5)
Feeling slowed down ^2^	12 (1.1)	Sadness ^2^	5 (0.5)
Difficulty concentrating ^2^	11 (1.0)	Erratic behavior	3 (0.3)
“Don’t feel right” ^2^	7 (0.7)	Delirious	2 (0.2)
Acting funny or different	6 (0.6)	Mental status change	1 (0.1)
Behavior change	4 (0.4)	More lively	1 (0.1)
Don’t recognize things	3 (0.3)	Stressed out	1 (0.1)
Cannot do/lost interest in normal tasks	3 (0.3)	Want to hurt self	1 (0.1)
Cognitive change	3 (0.3)	Feel lost	1 (0.1)
Trouble understanding things	2 (0.2)	State of shock	1 (0.1)
Trouble learning things	1 (0.1)	Make poor judgment	1 (0.1)
Repeating same things	1 (0.1)		

Note: 53 participants responded that they did not know any concussion symptoms. ^1^ Data obtained from the following question: In the space below, list what you think are the signs and symptoms of a concussion.” (Participants were then asked to answer in a free-response text-box). ^2^ Symptoms also appeared in symptom knowledge scale.

**Table 3:** Comparison of concussion symptoms reported by parents of middle school students (n = 1062).

Symptoms	Identified on Free-Response Item ^1^, n (%)	Identified “Yes” on Scale Measure ^2^, n (%)	Counts of Symptoms per Measure				McNemar Odds Ratio (95% CI) ^3^
			Both	Free-Response Item Only	Scale Measure Only	Neither	
Headache	526 (49.5)	877 (82.6)	481	45	396	140	0.11 (0.08, 0.15) *
Dizziness	472 (44.4)	958 (90.2)	450	22	508	82	0.04 (0.03, 0.07) *
Nausea or vomiting	297 (28.0)	833 (78.4)	282	15	551	214	0.03 (0.02, 0.05) *
Confusion	229 (21.6)	905 (85.2)	214	15	691	142	0.02 (0.01, 0.04) *
Blurred vision	175 (16.5)	928 (87.4)	168	7	760	127	0.009 (0.004, 0.02) *
Fatigue or low energy	151 (14.2)	655 (61.7)	131	20	524	387	0.04 (0.02, 0.06) *
Difficulty remembering	149 (14.0)	861 (81.1)	134	15	727	186	0.02 (0.01, 0.03) *
Balance problems	37 (3.5)	917 (86.4)	34	3	883	142	0.003 (0.001, 0.01) *
Sensitivity to light	34 (3.2)	698 (65.7)	32	2	666	362	0.003 (0.001, 0.01) *
Drowsiness	31 (2.9)	803 (75.6)	26	5	777	254	0.006 (0.003, 0.02) *
Feeling like “in a fog”	12 (1.1)	811 (76.4)	10	2	801	249	0.003 (0.001, 0.01) *
Feeling slowed down	12 (1.1)	675 (63.6)	9	3	666	384	0.005 (0.001, 0.01) *
Difficulty concentrating	11 (1.0)	845 (79.6)	8	3	837	214	0.004 (0.01, 0.01) *
“Don’t feel right”	7 (0.7)	805 (75.8)	5	2	800	255	0.003 (0.001, 0.01) *
Neck pain	7 (0.7)	654 (61.6)	7	0	647	408	N/A
Irritability	7 (0.7)	464 (43.7)	4	3	460	595	0.007 (0.002, 0.02) *
Nervous or anxious	5 (0.5)	295 (27.8)	2	3	374	683	0.008 (0.003, 0.03) *
Sadness	5 (0.5)	376 (35.4)	1	4	294	763	0.01 (0.005, 0.04) *
Sensitivity to noise	4 (0.4)	577 (54.3)	3	1	574	484	0.002 (0.001, 0.01) *
“Pressure in the head”	4 (0.4)	808 (76.1)	1	3	807	251	0.004 (0.001, 0.01) *
Trouble falling asleep	0	387 (36.4)	0	0	387	675	N/A
More emotional	0	365 (34.4)	0	0	365	697	N/A

* Denotes statistical significance (i.e., 95% CI excluded 1.00). ^1^ Data obtained from the following question: In the space below, list what you think are the signs and symptoms of a concussion.” (Participants were then asked to answer in a free-response text-box). ^2^ Data obtained from the following question: “Do you think the following are signs and symptoms of a concussion?” (Participants were given option of responding with “Yes”, “Maybe”, or “No”). **^3^** McNemar odds ratios examined discordance between noting symptoms on the free-response item versus the scale measures. The odds ratios were calculated as the number of participants identifying a symptom on free-response item only, divided by the number of participants identifying a symptom (i.e., responding “Yes”) on the scale measure only.

**Table 4:** Concussion symptoms reported by parents of middle school (MS) students (n = 1062) via free-response item, by sample characteristics.

Symptom Reported	n	Sex			Age (in Years)		
		Male	Female	*p*-Value	<40	≥40	*p*-Value
Headache	526	166 (45.6)	360 (51.6)	0.06	224 (43.8)	302 (54.9)	<0.001 *
Dizziness	472	155 (42.6)	317 (45.4)	0.38	203 (39.6)	269 (48.9)	0.002 *
Nausea or vomiting	297	74 (20.3)	223 (31.9)	<0.001 *	130 (25.4)	167 (30.4)	0.07
Confusion	229	70 (19.2)	159 (22.8)	0.18	113 (22.1)	116 (21.1)	0.70
Blurred vision	175	55 (15.1)	120 (17.2)	0.39	73 (14.3)	102 (18.5)	0.06
Fatigue or low energy	151	31 (8.5)	120 (17.2)	<0.001 *	71 (13.9)	80 (14.5)	0.75
Difficulty remembering	149	67 (18.4)	82 (11.7)	0.003 *	77 (15.0)	72 (13.1)	0.36
Loss of consciousness	149	49 (13.5)	100 (14.3)	0.70	76 (14.8)	73 (13.3)	0.46
Eyes/pupils do not look right	104	24 (6.6)	80 (11.5)	0.01 *	34 (6.6)	70 (12.7)	<0.001 *
Feel sleepy	98	17 (4.7)	81 (11.6)	<0.001 *	42 (8.2)	56 (10.2)	0.27
**Symptom Reported**	**n**	**Race/Ethnicity (Identified as White/Non-Hispanic)**			**Highest Level of Education Completed (Bachelor’s Degree or More)**		
		**Yes**	**No**	***p*-Value**	**Yes**	**No**	***p*-Value**
Headache	526	331 (52.9)	195 (44.7)	0.009 *	236 (49.8)	290 (49.3)	0.88
Dizziness	472	281 (44.9)	191 (43.8)	0.73	214 (45.1)	258 (43.9)	0.68
Nausea or vomiting	297	209 (33.4)	88 (20.2)	<0.001 *	134 (28.3)	163 (27.7)	0.84
Confusion	229	155 (24.8)	74 (17.0)	0.002 *	111 (23.4)	118 (20.1)	0.19
Blurred vision	175	123 (19.6)	52 (11.9)	<0.001 *	76 (16.0)	99 (16.8)	0.73
Fatigue or low energy	151	96 (15.3)	55 (12.6)	0.21	74 (15.6)	77 (13.1)	0.24
Difficulty remembering	149	77 (12.3)	72 (16.5)	0.052	78 (16.5)	71 (12.1)	0.04 *
Loss of consciousness	149	82 (13.1)	67 (15.4)	0.30	72 (15.2)	77 (13.1)	0.33
Eyes/pupils do not look right	104	80 (12.8)	24 (5.5)	<0.001 *	45 (9.5)	59 (10.0)	0.77
Feel sleepy	98	65 (10.4)	33 (7.6)	0.12	29 (6.1)	69 (11.7)	0.002 *
**Symptom Reported**	**n**	**Parent or MS Child** **Reported Concussion History**			**MS Child Played Organized Sports within Past Year**		
		**Yes**	**No**	***p*-Value**	**Yes**	**No**	***p*-Value**
Headache	526	180 (48.9)	346 (49.9)	0.77	393 (49.9)	133 (48.5)	0.70
Dizziness	472	158 (42.9)	314 (45.2)	0.47	363 (46.1)	109 (39.8)	0.07
Nausea or vomiting	297	111 (30.2)	186 (26.8)	0.25	223 (28.3)	74 (27.0)	0.68
Confusion	229	84 (22.8)	145 (20.9)	0.47	179 (22.7)	50 (18.2)	0.12
Blurred vision	175	67 (18.2)	108 (15.6)	0.27	129 (16.4)	46 (16.8)	0.87
Fatigue or low energy	151	62 (16.8)	89 (12.8)	0.07	112 (14.2)	39 (14.2)	0.99
Difficulty remembering	149	48 (13.0)	101 (14.6)	0.50	119 (15.1)	30 (10.9)	0.09
Loss of consciousness	149	54 (14.7)	95 (13.7)	0.66	110 (14)	39 (14.2)	0.91
Eyes/pupils do not look right	104	43 (11.7)	61 (8.8)	0.13	78 (9.9)	26 (9.5)	0.84
Feel sleepy	98	33 (9.0)	65 (9.4)	0.83	98 (9.2)	71 (9.0)	0.68

Note: *p*-values calculated from chi-square tests; * indicated *p*-value < 0.05.

## Data Availability

The data available in this study are available upon request to the corresponding author.
